# One Health antimicrobial resistance modelling: from science to policy

**DOI:** 10.1016/j.soh.2026.100146

**Published:** 2026-01-10

**Authors:** Carys J. Redman-White, Gwen Knight, Cristina Lanzas, Rodolphe Mader, Bram van Bunnik, Fernando O. Mardones, Adrian Muwonge, Guillaume Lhermie, Andrew R. Peters, Dominic Moran

**Affiliations:** a Global Agriculture and Food Systems, The Royal (Dick) School of Veterinary Studies, University of Edinburgh, Easter Bush Campus, Roslin, EH25 9RG, United Kingdom; b The Digital One Health Laboratory, The Roslin Institute, The Royal (Dick) School of Veterinary Studies, University of Edinburgh, Easter Bush Campus, Roslin, EH25 9RG, United Kingdom; c Antimicrobial Resistance Centre, London School of Hygiene and Tropical Medicine, London, WC1E 7HT, United Kingdom; d Department of Population Health and Pathobiology, College of Veterinary Medicine, North Carolina State University, CVM Terry Center, Raleigh, NC, 27606, United States; e International Centre for Antimicrobial Resistance Solutions, 2300, Copenhagen S, Denmark; f Epidemiology Economics and Risk Assessment, The Roslin Institute, The Royal (Dick) School of Veterinary Studies, University of Edinburgh, Easter Bush Campus, Roslin, EH25 9RG, United Kingdom; g School of Veterinary Medicine, Pontificia Universidad Católica de Chile, 7820436, Macul, Región Metropolitana, Chile; h School of Public Policy, University of Calgary, Calgary, AB, T2P 1H9, Canada; i College of Veterinary Medicine, University of Calgary, Calgary, AB, T2N 4Z6, Canada; j The Royal (Dick) School of Veterinary Studies, University of Edinburgh, Easter Bush Campus, Roslin, EH25 9RG, United Kingdom

**Keywords:** Antimicrobial resistance, One Health, Models, Policy

## Abstract

Modern human and veterinary medical interventions to combat infectious diseases depend on the continued efficacy of antimicrobial drugs. Antimicrobial resistance (AMR) is the quintessential One Health challenge threatening human and animal health and welfare and has environmental effects on ecological communities in soil and water. Policy guidance on AMR needs to anticipate the likely outcomes of different interventions and courses of action. For that, transdisciplinary collaboration to understand the development, spread, and impacts of AMR is crucial. We report the outcomes of an international workshop that explored the challenges and opportunities for modelling AMR across One Health settings. They include the disparity of data quality and availability, the broader knowledge gaps in key areas such as the relationship between antimicrobial use (AMU) and AMR, and the difficulty of defining AMR as a single outcome given its heterogeneity. Differences between microbial species, resistance genes, environments (i.e., terrestrial vs. aquatic) and practical settings (e.g., human clinical vs. veterinary, or individual vs. population) complicate the generalizability of model applications. However, synoptic AMR metrics are necessary to cut through the complexity for policymaking. We discuss the status of AMR modelling with respect to a hierarchy of modelling evidence for decision-making. Finally, we consider learnings from modelling other wicked environmental challenges to develop a pragmatic approach to inform policy.

## 1 Introduction

Antimicrobial resistance (AMR) is a global One Health challenge. Antimicrobials are vital to human and veterinary medicine. To conserve their effectiveness, we need to clarify the interactions between causes and consequences of antimicrobial exposure and AMR in human, veterinary, crop production, and environmental settings [1]. Alongside this, a system of governance and stewardship must be developed to align with the common property and market failure dimensions of AMR, and the concept of antimicrobial susceptibility as a global public good accessed whenever antimicrobials are used. These combined challenges make AMR a wicked problem [2]. Moreover, gaps in important data and governance hinder translation of science into effective policy. Modelling, using mathematical representations of the system, has important applications for developing mechanistic understanding of AMR and informing policy. Computational modelling is used to design, implement, and evaluate policies. Models help to integrate existing data to formalize the processes driving the system, project scenarios, and to evaluate policies [3]. This narrative-methodological review considers alternative modelling approaches and highlights interdisciplinary and transdisciplinary challenges faced in using modelling to prioritize effective and economically efficient interventions to mitigate antimicrobial exposure and AMR. We also consider the scope for learning from other planetary health challenges that offer alternative perspectives on some dimensions of the problem, notably climate change [4,5]. The content emerged from discussions at a symposium funded by the Organisation for Economic Co-operation and Development (OECD) Cooperative Research Programme on Sustainable Agricultural and Food Systems, at which experts from different disciplines considered the modelling interface between food animals and humans in clinical and community contexts. The discussion highlighted how different disciplinary perspectives may lead to complementary pragmatic policy options.

This paper is structured as follows. Section one considers attributes of the AMR problem as both a scientific and a public policy challenge. Section two considers challenges occurring at each level of a modelling hierarchy. Section three considers what we need from models to develop evidence-based policy, including what can be learnt from climate change mitigation. Finally, we offer some pragmatic conclusions on model development needs.

## 2 A wicked problem for science and public policy

Despite mounting evidence of impending global health and economic burdens, AMR is arguably failing to gain sufficient policy traction for several reasons. In contrast to acute health crises, AMR impacts tend to be cumulative, disparate, and less visible. Rather than deriving from a single pathogen, AMR involves a diversity of microbes with varied ecological, epidemiological, and pharmaceutical implications, so-called bug-drug-context combinations [6]. This heterogeneity and complexity complicate communication and undermine the engagement of publics and governments [7]. Scrutiny of the causes and effects reveals a complex cocktail of origins and causalities. Typically, there is an asymmetry in the incidence of costs and benefits of intervening that leads to inertia around actions to curb antimicrobial use and to minimize transmission of resistant organisms and genetic material.

There are many data gaps to address to guide effective policy interventions. For example, even estimating global antimicrobial use (AMU) relies on layers of inference [8]. Such basic data gaps complicate the assessment of interventions, as in many cases baseline data are either unavailable or highly biased. Surveillance to estimate, for instance, resistance prevalence and AMR profiles of isolated pathogens can also be costly [9].

AMU, specifically in medical and agricultural sectors, is a known and modifiable risk factor for AMR, and reducing AMU is the cornerstone of current AMR policies [10]. However, in addition to quantification, the AMU-AMR relationship is poorly understood and heterogeneous across bug-drug combinations. Additionally, resistance is shaped by selection through both AMU and co-selection by other substances such as biocides, transition metals and metalloids, as well as transmission of pathogens and AMR genes (ARGs). For a single bug-drug combination, ARGs may have varied fitness impacts, positive and negative [11], affected not only by presence of antimicrobials, but other environmental selective agents. The differences in mechanisms, spectrum of action, cross-resistance, and other variations complicate the characterization of AMR as a monolith. Transmission of resistant organisms and ARGs, including horizontal gene transfer, must be understood to appreciate the relative importance of selection and transmission and target interventions. Asymptomatic colonization with resistant organisms in different settings also requires further elucidation.

The relative magnitudes and directions of AMU-AMR interactions between humans, other animals, and the environment must be understood to guide interventions. The degree to which AMR in humans is attributable to AMU in livestock production and aquaculture is contested, and while animal health has both intrinsic and economic value, the attribution of AMU in animal-source food production to AMR in humans is central to many policy decisions. The terrestrial and aquatic environments, despite potentially having great significance to AMR selection and transmission, are frequently overlooked, both in modelling and in broader research and policy [12,13].

Given these uncertainties, it is unsurprising that there is currently no appraisal framework to illustrate costs and benefits of interventions in any one country, let alone as a basis for international cooperation. National AMR Action Plans are often under-resourced and held back by governance and infrastructure limitations [14,15]. However, other global environmental challenges could provide pointers to the management of AMR, and to the complexity of combining different stakeholder needs and perspectives. Climate change mitigation in particular illustrates how countries and states can address a similarly wicked problem using integrated assessment modelling and approaches that clarify where to intervene, considering the relative effectiveness and net benefits of interventions. Allocating scarce resources to optimal AMR mitigation strategies is the ultimate transdisciplinary objective for researchers modelling AMR.

## 3 Modelling challenges

To cut through some of the biophysical complexity of the problem, policy evidence will inevitably rely on modelling of both AMR itself and the AMU-AMR link. Statistical modelling using data from observational and intervention studies is crucial for informing our collective understanding. However, the interplay of heterogeneous scales and current data gaps make mathematical modelling the most pragmatic approach to explore the complexity of AMR. Several challenges have been identified affecting a wide range of types of AMR models [16,17].

Models can take a top-down or a bottom-up approach in terms of scale: focusing on policy at an international, national or regional scale, or strategies implemented by farms or individuals. They may focus on epidemiological, economic, agricultural or other aspects of AMR, and may be AMR-specific or adopt a broader AMR-sensitive perspective [12]. A common thread is that heterogeneity and knowledge gaps regarding the relationship between AMU and AMR make a central component of many models extremely difficult to characterize. Similarly, key parameters remain unknown; for example, the relative importance of different transmission pathways across human, animal, and environmental settings. This is crucial both for quantifying components of One Health models and prioritizing focus in models with narrower scope. Data gaps in these areas are necessarily filled by many model assumptions. Even in developing the most parsimonious models, such assumptions cumulate, potentially increasing uncertainty in model outputs. Sensitivity analysis is key to testing the robustness of conclusions, but systematic analysis suggests this is lacking, with 40 % of population-level AMR models including no parameter sensitivity or uncertainty analysis [12]. The TRACE modelling guidelines were established in 2010 to promote good practice in development, testing, and communication of mathematical models [18]. However, a systematic review of mathematical models of AMR found none that met these guidelines [17].

**Fig. 1:**
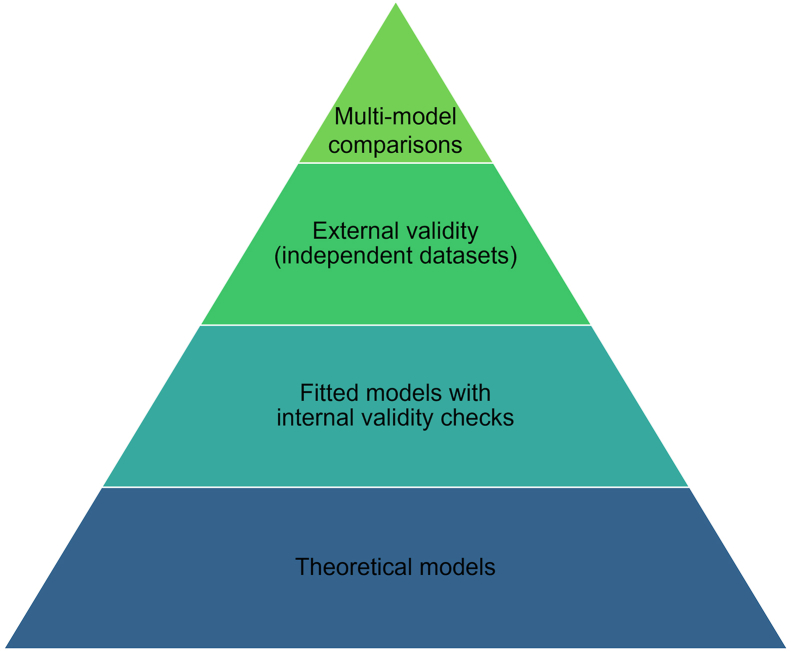
Hierarchy of mathematical modelling evidence.

Models classify hierarchically according to their ability to match exact dynamics (Fig. 1). Theoretical models are at the base and their utility lies in exploring potential mechanisms and scenarios. Next are models fitted to observed data with internal validity checks, and above these are models validated externally with independent datasets. At the top are multi-model comparisons, the modelling equivalent of a meta-analysis, including planning, conducting and reporting comparisons of AMU-AMR and related interventions in a systematic and structured manner for supporting policy decisions [19].

Population-level models predominate amongst published mathematical models of AMR [17]. These include a range of modelling techniques, but recent systematic reviews indicate that the literature focuses overwhelmingly on humans [12,17]. An analysis of 273 population-level models found that 89 % considered only humans, 7 % modelled an animal population, and 2 % modelled AMR in plant populations. Two percent of models addressed human-animal combined populations, and none included all of human, animal and environmental or plant populations. Nine percent of these models included an economic cost-benefit component [12]. Evidently there is room for more transdisciplinary, and One Health approaches; the same review showed a bias towards investigation of bacterial AMR, accounting for 63 % of the models included in the review [12].

### 3.1 Data quality and availability

Data availability for statistical modelling varies widely between countries, and those relating to AMR are incomplete and often siloed, poorly harmonized, and difficult to access in many countries [20]. Surveillance challenges include deciding which microbial species and drugs to monitor, and which metrics and techniques to apply with limited resources. Moreover, most AMR data is from routine surveillance used to inform clinical care rather than to give prevalence estimates, and hence is often biased toward those failing empiric antibiotic therapy, the most critically ill patients and samples from a limited set of infections.

Systematic reviews and meta-analyses are important tools for bringing together published evidence, and improved methods can automate aspects of systematic review, allowing researchers to focus on the analysis aspect. But source studies must be well-designed and reported, with open access to data and code. While automated tools can facilitate recovery of results data from graphs, this is far from ideal.

Statistical modelling, as well as making sense of observed data, can inform mathematical modelling by narrowing parameter space. For example, phylodynamic analysis of genomic data from bacterial strains in the presence and absence of antimicrobials can provide estimates of relative fitness of ARGs. So far, such analyses have observed marked variation in ARG fitness costs even for a single “bug-drug combination,” emphasizing the hazard of generalization [11].

### 3.2 Theoretical models

Theoretical models, meaning those modelling phenomena more broadly, rather than representing a specific system, can be applied to numerous aspects of AMR, including exploring potential impacts of interventions and other contextual changes.

**Fig. 2:**
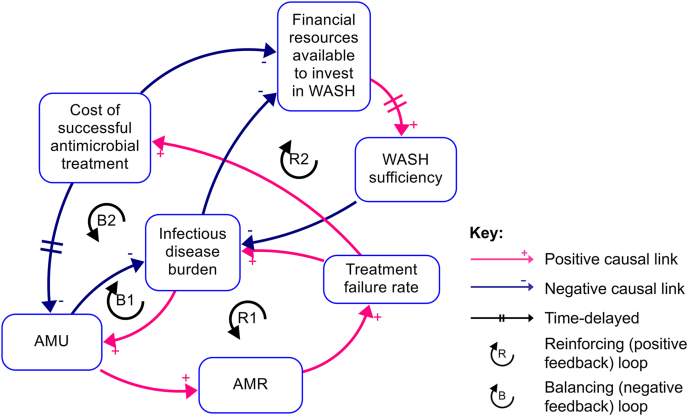
An example of CLD depicting relationships between AMU, AMR, and WASH. Arrows depict causal relationships between variables, with “+” indicating variables that change in the same direction and “−” indicating variables that change in the opposite direction. Delayed causal relationships are depicted by two perpendicular lines. The two reinforcing (positive feedback) and two balancing (negative feedback) loops are labelled. Abbreviations: CLD, causal loop diagram; AMU, antimicrobial use; AMR, antimicrobial resistance; WASH, water, sanitation and hygiene.

Some systems modelling approaches may fall in this category. For example, potential AMR-sensitive and specific interventions can be identified by system mapping using causal loop diagrams (CLDs) (Fig. 2). CLDs can form the basis of quantitative system dynamics models, although characterizing and quantifying the links to build and validate these is challenging. Economic modelling, including farm scale optimization or the demand response to antimicrobial pricing and taxation, can also be used to explore potential AMU policy strategies.

Microbiological and epidemiological aspects can also be addressed with theoretical models. For instance, the use of antimicrobials for metaphylaxis in livestock production and aquaculture, which has been debated due to their mass consumption of antimicrobials, can be evaluated [21]. Relatively simple differential equation models provide a way to investigate even when parameters are unknown by exploring a wide parameter space. In one model, the fractions of human and food animal hosts carrying resistant bacteria were each represented as a single variable, influenced by use of antibiotics, reversion of bacteria to susceptible wild-type, and bidirectional transmission of resistant bacteria between humans and livestock. The authors explore the model's behavior under a wide range of conditions, concluding that even completely ceasing antibiotic use in livestock would not substantially decrease resistance in human hosts [22].

### 3.3 Fitted models with internal validity checks

Key difficulties with constructing fitted models are the need for appropriate data and the heterogeneous nature of AMR. The data needed to parameterize and test the models depends on their purpose. It may be more difficult to obtain the data needed to model AMR in microbial commensals than in clinical cases, as much of the available AMR data derives from diagnostics, at least in the human setting. Due to AMR heterogeneity, models must either be highly specific or sufficiently complex to account for differences among bug-drug pairs, ARGs, microbial strains and overall settings. For example, a model of AMR dynamics in *Escherichia coli* bacteria might need to account for the fact that this bacterial species has many subtypes with different niches, as well as scope for horizontal gene transfer and multiple resistance mechanisms.

### 3.4 Models with external validation (independent datasets)

External validation using independent datasets requires a further dataset that is genuinely compatible with, and comparable to, the dataset used to develop the model. Hellweger (2013) [23] uses data from broader literature and measurements from a specific study site to parameterize a mechanistic model of AMR co-selection. The author runs simulations for three hypotheses for environmental co-selection for tetracycline resistance, comparing these to observed resistance patterns to assess hypothesis plausibility. The availability of data for external validation is a barrier to improved AMR modelling [17] and makes this extremely difficult to achieve in some settings. One strategy to address this is to split a single, sufficiently large dataset so that one section of the dataset is used for training the model and the rest is reserved for external validation.

### 3.5 Multi-model comparisons

Multi-model comparisons have been used for pathogen-specific infectious disease challenges such as COVID-19 [24,25] but the heterogeneity of AMR and the lack of a critical mass of comparable models make a multi-model comparison currently unfeasible. Such approaches require harmonization of parameters and models, which must be chosen for their ability to answer a specific research question [19]. AMR occurs in a myriad of microbes and settings, for which parameters may vary markedly [26]; realistically, multi-model comparisons would likely have to focus on a specific set of conditions, for example one bug-drug-context combination.

## 4 Modelling for policy

The modelling complexity explains some of the inertia around a clear suite of interventions to mitigate AMR. Alternative problem frames could guide AMR policy, particularly those from environmental policy targeting diffuse pollution.

Human activity is stressing planetary boundaries, defined by pollutant concentration limits and levels of resource extraction. The impact of AMU on microbial communities can be similarly framed, with antimicrobial susceptibility considered as a depletable common-pool resource, or antimicrobials and ARGs as pollutants [27]. These framings can imply specific modelling challenges to inform policies on drug access or rationing, or the use of pricing mechanisms (e.g., taxation) of the notional external cost associated with excessive AMU [28].

### 4.1 A climate change mitigation parallel

A pollution mitigation frame allows us to learn from approaches to the similarly wicked challenge of greenhouse gas (GHG) emissions mitigation, and to some extent adaptation to climate change. Identification and prioritization of cost-effective emissions mitigation interventions have been informed by pollution abatement theory that seeks to prioritize among technically effective interventions. This prioritization leads to the development of abatement cost curves that guide policy across all emitting sectors. Cost-effectiveness is a vital attribute to a feasible and effective mitigation policy, but no similar framework has been considered to prioritize AMU or AMR interventions: policy is being enacted without evidence to judge the economic efficiency. This shortcoming is partly due to the modelling challenges outlined here, but even for relatively certain AMU interventions, there is no clear picture depicting relative cost-effectiveness in different settings.

Extending the climate change analogy, it is possible to consider some AMR “pollution” as an inevitable cost of unavoidable AMU in clinical and veterinary medicine. Consequently, the world still needs to adapt, not only in terms of curbing antimicrobial demand, but more obviously in terms of accelerating discovery of new pharmaceutical or other AMR-sensitive healthcare interventions. In either case, a clearer view of costs and benefits of action and inaction will help to develop some consensus on policy options.

A final climate change analogy and modelling challenge is the conceptualization of a potential social cost or shadow price of AMR, akin to the notional social cost of carbon emissions (SCC), representing the global damage cost of an extra unit of emissions. The SCC is a key metric for cost-benefit decision-making regarding emissions reduction and is estimated from integrated assessment models linking atmospheric GHG concentrations with global damage costs [29]. The scientific basis for much of this evolving modelling architecture resides with the Intergovernmental Panel on Climate Change (IPCC), which cumulates knowledge to address policy questions around emissions mitigation. A similar modelling collaboration is conspicuously absent in the AMR sphere, meaning that there is currently no coherent integrated modelling architecture developing a national or international cost-benefit framing of the AMR challenge. Addressing this gap represents an important opportunity for the Independent Panel on Evidence for Action against Antimicrobial Resistance (IPEA) [30], currently under negotiation and consultation by the UN Quadripartite Group on AMR (World Health Organization/Food and Agriculture Organization of the United Nations/World Organisation for Animal Health/United Nations Environment Programme).

### 4.2 Future directions

Modelling can contribute both to the scientific understanding of AMR and to development of appropriate policies for its mitigation. AMR and the relative roles of processes that influence and are influenced by it are clearly highly heterogeneous. “AMR” as a discrete entity and its drivers are difficult to quantify when different ARGs in different microbial species are under different selection pressures in different locations and may have different implications for those exposed to resistant microbes. The need to balance the global nature of AMR with numerous context-specific elements makes this particularly challenging. Transdisciplinary and international modelling collaborations will be key to addressing this challenge, particularly for addressing AMR at a global scale.

Systematic reviews have identified specific research questions, while emphasizing the importance of rigorous study design and reporting, including data and code access. Data harmonization is necessary to compare datasets; a particular challenge given the variety of phenotypic, genetic, whole-genome and metagenomic methods for measuring AMR, the often-biased populations from whom the AMR samples are taken and indicators to measure AMU. For phenotypic assays, further consideration must be given to which species will be assessed for resistance, and to which antimicrobials. Similarly, specific ARGs to be quantified using genetic methods must be selected from previously characterized genes. Sampling strategies also affect the data produced, with ongoing debate over the optimal sampling strategy. Due to the structure of most surveillance programs in the human sector, infection samples are overrepresented among AMR data, while in the animal sector, surveillance systems are often primarily established in a food safety perspective, with sampling occurring in healthy animals at slaughter. Tools such as a Digital One Health framework [31] represent one approach to maximize efficiency and harmonization of surveillance. In scientific publishing, transparency of study data and modelling code are necessary to make optimal use of resources as well as ensuring reproducibility, and at present are under-implemented [14].

## 5 Conclusion

The nature of AMR as a largely invisible pandemic is a challenge in terms of motivating sufficiently urgent policy responses compared to more conspicuous planetary health challenges. The One Health paradigm emphasizes the interconnectedness of settings that have largely been analyzed separately. However, modelling efforts are needed to reconcile analytical scales to address the key transdisciplinary challenge: how to reduce AMR cost-effectively. Modelling evidence gaps outlined here illustrate significant challenges with data gathering, model communication, and arguably an overdue conversation on ultimate modelling objectives. Development of a focused modelling architecture needs to involve more disciplines than is currently the case. Integrated modelling of AMR is still in its infancy, with gaps in knowledge and data availability, and limited discussion of how models combine to address key policy questions. Consequently, evidence is unconvincing to mobilize political attention and thus resources. We contend that a more concerted transdisciplinary modelling effort is required to generate the political will to take the necessary steps to manage this planetary health crisis.

## CRediT authorship contribution statement

**Carys J. Redman-White:** Writing – review & editing, Writing – original draft, Project administration, Funding acquisition, Conceptualization. **Gwen Knight:** Writing – review & editing, Writing – original draft. **Cristina Lanzas:** Writing – review & editing, Writing – original draft, Visualization. **Rodolphe Mader:** Writing – review & editing, Writing – original draft. **Bram van Bunnik:** Writing – review & editing, Writing – original draft. **Fernando O. Mardones:** Writing – review & editing, Writing – original draft. **Adrian Muwonge:** Writing – review & editing, Writing – original draft, Supervision. **Guillaume Lhermie:** Writing – review & editing. **Andrew R. Peters:** Writing – review & editing, Supervision. **Dominic Moran:** Writing – review & editing, Writing – original draft, Supervision, Project administration, Funding acquisition, Conceptualization.

## Funding

The authors acknowledge support from the OECD Co-operative Research Programme: Sustainable Agricultural and Food Systems. DM also acknowledges support under UKRI awards BB/T004436/1 and BB/T004452/1. CRW acknowledges support for a studentship part-funded by Zoetis and the UKRI Biotechnology and Biological Sciences Research Council (BBSRC) (grant number BB/T00875X/1). CL also acknowledges support by the Centres for Disease Control and Prevention (CDC) (grant number U01CK000587) and the US National Institutes of Health (NIH) (grant number R35GM134934). FOM acknowledges support by the FONDECYT Regular N° 1241544, funded by Agencia Nacional de Investigación y Desarrollo (ANID, Chile). GMK was supported by Medical Research Council UK (grant number MR/W026643/1).

## Declaration of competing interest

The authors declare no competing interests.
